# mD-UPLC-MS/MS: Next Generation of mAb Characterization by Multidimensional Ultraperformance Liquid Chromatography-Mass Spectrometry and Parallel On-Column LysC and Trypsin Digestion

**DOI:** 10.1021/acs.analchem.1c04450

**Published:** 2022-05-12

**Authors:** Saban Oezipek, Sina Hoelterhoff, Simon Breuer, Christian Bell, Anja Bathke

**Affiliations:** Pharma Technical Development, F. Hoffmann-La Roche, Grenzacherstrasse 124, 4070 Basel, Switzerland

## Abstract

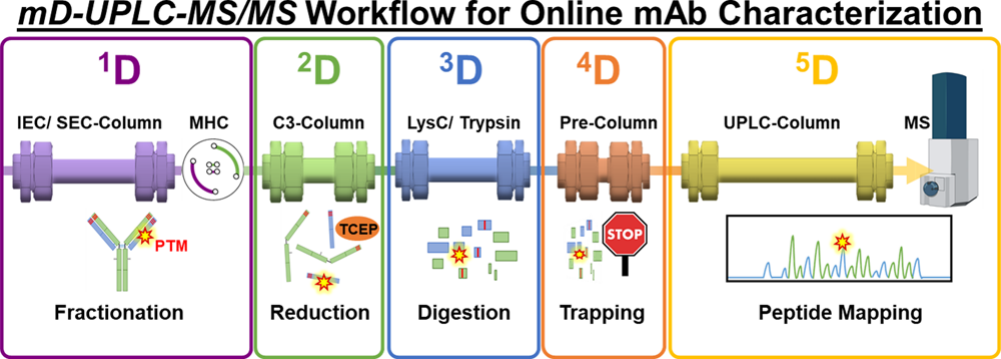

For the past few years, multidimensional liquid chromatography-mass spectrometry (LC-MS) systems have been commonly used to characterize post-translational modifications (PTMs) of therapeutic antibodies (mAbs). In most cases, this is performed by fractionation of charge variants by ion-exchange chromatography and subsequent online LC-MS peptide mapping analysis. In this study, we developed a multidimensional ultra-performance-liquid-chromatography-mass spectrometry system (mD-UPLC-MS/MS) for PTM characterization and quantification, allowing both rapid analysis and decreased risk of artificial modifications during sample preparation. We implemented UPLC columns for peptide mapping analysis, facilitating the linkage between mD-LC and routine LC-MS workflows. Furthermore, the introduced system incorporates a novel in-parallel trypsin and LysC on-column digestion setup, followed by a combined peptide mapping analysis. This parallel digestion with different enzymes enhances characterization by generating two distinct peptides. Using this approach, a low retentive ethylene oxide adduct of a bispecific antibody was successfully characterized within this study. In summary, our approach allows versatile and rapid analysis of PTMs, enabling efficient characterization of therapeutic molecules.

## Introduction

Therapeutic monoclonal antibodies (mAbs) have become increasingly important for the treatment of critical diseases, therefore, for the pharmaceutical industry.^1^ To ensure patient safety, it is crucial that quality control confirms the reliability and consistency of pharmaceutical biotech products across the entire product life cycle. For this purpose, protein stability is a key factor that has to be maintained from production until application to assure a safe and efficacious treatment of patients.^2−4^ To provide sufficient quality of biopharmaceutical products, the U.S. Food and Drug Administration (FDA) recommends the characterization and monitoring of critical quality attributes (CQAs) directly at the peptide level.^5,6^ To comply with the requirements, peptide mapping analysis has become a standard method for characterizing the primary structure of biopharmaceuticals and thus the accurate identification of post-translational modifications (PTMs).^5,7,8^ Nevertheless, this analysis requires a labor-intense and time-consuming manual sample preparation.^9^ To increase efficiency, two approaches toward method automation were established. On the one hand, manual sample preparation can be automated with pipetting robots, allowing simultaneous processing of multiple samples in 96-well plates.^10^ On the other hand, automated sample preparation and peptide mapping analysis can be accomplished by liquid chromatography (LC)-based methods, where the sample is injected directly into the multidimensional LC system (mD-LC) and the analyte is online processed and analyzed.^11^ With LC-based methods, only one sample at a time is processed; hence, this method is particularly suitable for a smaller number of samples compared to the automation by pipetting robots. However, the key advantage is that this approach can be combined with chromatographic methods, respectively, dimensions (e.g., ion-exchange chromatography (IEC), size-exclusion chromatography (SEC), Protein-A) prior peptide mapping.^11−14^ The additional dimension opens up a wide range of possibilities for system expansion and specific applications within the pharmaceutical industry. Due to the ability to fractionate and characterize peaks of interest, this approach is well suited for the extended characterization of mAbs to deepen the knowledge and support the analytical method development and product characterization.^15^ Therefore, this approach is especially applicable for early and late-stage mAb development. For the analysis and characterization of mAb degradation products (e.g., asparagine, deamidation, methionine oxidation, lysine glycation), Gstöttner et al. (2018) developed a multidimensional LC system (mD-LC) coupled to a high-resolution mass spectrometer. The developed four-dimensional high performance liquid chromatography–mass spectrometry (4D HPLC/MS) system incorporates an ion-exchange chromatography (IEC) as the first dimension (^1^D) and allows online fractionation of charge variants using a multiple heart cutting valve (MHC) from Agilent Technologies. The subsequent three dimensions after fractionation are directly used for online sample preparation and peptide mapping prior to MS-analysis (^2^D = reduction,^3^D = trypsin digestion,^4^D = peptide mapping). As Gstöttner et al. (2018) have shown, the characterization of five charge variants using the developed 4D-HPLC/MS method can be performed about 5.8 times faster than manual characterization (online 9 h vs offline 52 h), highlighting the efficiency of this automated LC-based approach. Nevertheless, the authors have critically reviewed the results and indicate that small, polar peptides (<1.3 kDa) are not retained in the trapping step, which results in a reduced sequence coverage (online: LC: 94%, HC: 86% vs offline: LC: 94%, HC: 94%). The loss of small, polar peptides while peptide mapping analysis can be critical, especially if they are declared as CQAs, severely limits the method and makes it less suitable.^14,15^ In this work, we present a novel approach to achieve increased sequence coverage and retention of small, polar peptides by introducing the latest evolution of our multidimensional LC-MS system, which we refer as an multidimensional-ultra-performance-liquid-chromatography-mass spectrometry (mD-UPLC-MS/MS) system. In addition, we show that our system enables the usage of long sub 2 μm UPLC columns for peptide mapping analysis through an optimized setup, which allows a system pressure up to 1300 bar.

Furthermore, the developed system supports a versatile digestion setup with either a single column (LysC/trypsin) or an in-parallel LysC and trypsin column setup.

## Experimental Section

### mD-UPLC-MS/MS Instrument Setup

The introduced mD-UPLC-MS/MS system is based on LC modules from Agilent Technologies (Waldbronn, Germany) coupled with the high-resolution mass spectrometer Impact II from Bruker Daltonics. Reagents for the analysis with the mD-UPLC-MS/MS instrument are listed in Supporting Table S1. The LC system is configured as two instruments within the OpenLab software package from Agilent Technologies, incorporating the modules listed in Supporting Table S2. For communication between the two instruments, a self-designed macro “valve event plugin” from ANGI (Gesellschaft für angewandte Informatik, Karlsruhe, Germany) was used. The macro starts the method of the second instrument and the mass spectrometer via a contact closure signal for each fraction in the first dimension.

### ^1^D Ion-Exchange Chromatography and Fractionation

The ^1^D method varies according to the product being analyzed and corresponds to the GxP method for IEC or SEC quality control (QC) analysis. For the characterization of Herceptin (trastuzumab) charge variants, a MAbPac WCX (4.0 × 250 mm, 10.0 μm) column from Thermo Fisher Scientific was employed as the first dimension. Unstressed Herceptin (150 μg) was injected into the system, and the parameters of Supporting Table S3 were chosen for the cation-exchange chromatography (CEX). By detecting the absorbance at 214 nm, the acidic, main, and basic peaks were fractionated with the MHC valve and stored in 120 μL loops of decks A and B. For reduction (^2^D), digestion (^3^D), trapping (^4^D), and peptide mapping analysis (^5^D), the fractions were subsequently processed with the following dimensions.

### ^2^D On-Column Reduction

The second dimension of the mD-UPLC-MS/MS instrument incorporates a Poroshell 300SB-C3 (2.1 × 12.5 mm, 5.0 μm) cartridge from Agilent Technologies for trapping and reduction of the ^1^D fractions. The fast on-column reduction was performed by flushing the trapped mAbs with 20 mM Tris-(2-carboxyethyl)-phosphin (TCEP). Afterward, the C3-Cartridge was washed and the reduced mAbs were eluted onto the immobilized enzyme reactor (IMER). The parameters and gradients of the second dimension are provided in Supporting Table S4.

### ^3^D On-Column Digestion

For online digestion of the reduced ^1^D fractions, a custom-made LysC (2.1 × 100 mm, Perfinity Biosciences) and/or trypsin (2.1 × 100 mm, Perfinity Biosciences) IMER was used as the third dimension. Thus, either an in-parallel or single enzymatic digestion setup can be selected with the mD-UPLC-MS/MS instrument. For the parallel setup, the flow is split in half in front of the columns and merged afterward by two T-pieces. This allows separated digestion with both columns and afterward the combined analysis of LysC and trypsin peptides. In addition, the mD-UPLC-MS/MS system allows a single enzymatic digestion setup where only one column is installed and the remaining ports of the T-pieces are blocked by stop plugs. For optimal digestion, the reduced ^1^D fractions are diluted with digestion buffer at a ratio of 1:6 with a biocompatible 100 μL of binary mixer from ASI-Analytical Scientific Instruments. During the digestion step, the IMER was connected in-line with the peptide trapping column and the flow-through digestion took approximately 70 s. A detailed description of the parameters can be found in Supporting Table S5.

### ^4^D Precolumn Trapping

After digestion, the eluting peptides were diluted with Milli-Q H_2_O at a ratio of 1:5.5 using a biocompatible 150 μL binary mixer from ASI-Analytical Scientific Instruments. The two dilution steps (^3^D,^4^D) result in a final acetonitrile (ACN) concentration of min. 1% for peptide trapping depending on the used precolumn. For the Herceptin analysis, an InfinityLab Poroshell 120 SB-C18 (3.0 × 5 mm, 1.9 μm) precolumn from Agilent Technologies was used. For the bispecific mAb (BsMAb) analysis, an ACQUITY UPLC BEH C18 VanGuard precolumn (2.1 × 5 mm, 1.7 μm) from Waters Corporation was incorporated into the system. After peptide trapping, the precolumn was washed and subsequently placed in-line with the analytical full-length UPLC column for peptide mapping analysis. The parameters of the fourth dimension are provided in Supporting Table S6.

### ^5^D Peptide Mapping Analysis

The peptide mapping analysis was initiated by switching the precolumn in-line with the analytical reversed-phase column. The used analytical column depends on the antibody to be investigated. For the Herceptin analysis, an InfinityLab Poroshell 120 SB-C18 (2.1 × 150 mm, 1.9 μm) column from Agilent Technologies was used. The chromatographic peptide separation for the BsMAb was performed using a UPLC BEH Peptide C18 column (2.1 × 150 mm, 1.7 μm) from Waters Corporation. The parameters and gradients are given in Supporting Table S7. For detection of MS1 and MS2 spectra, the high-resolution ESI-Q-ToF mass spectrometer Impact II from Bruker Daltonics was used. The mass spectrometer parameters are listed in Supporting Table S8.

## Results and Discussion

### Setup Differences of mD-UPLC-MS/MS vs 4D-HPLC/MS

**Figure 1:**
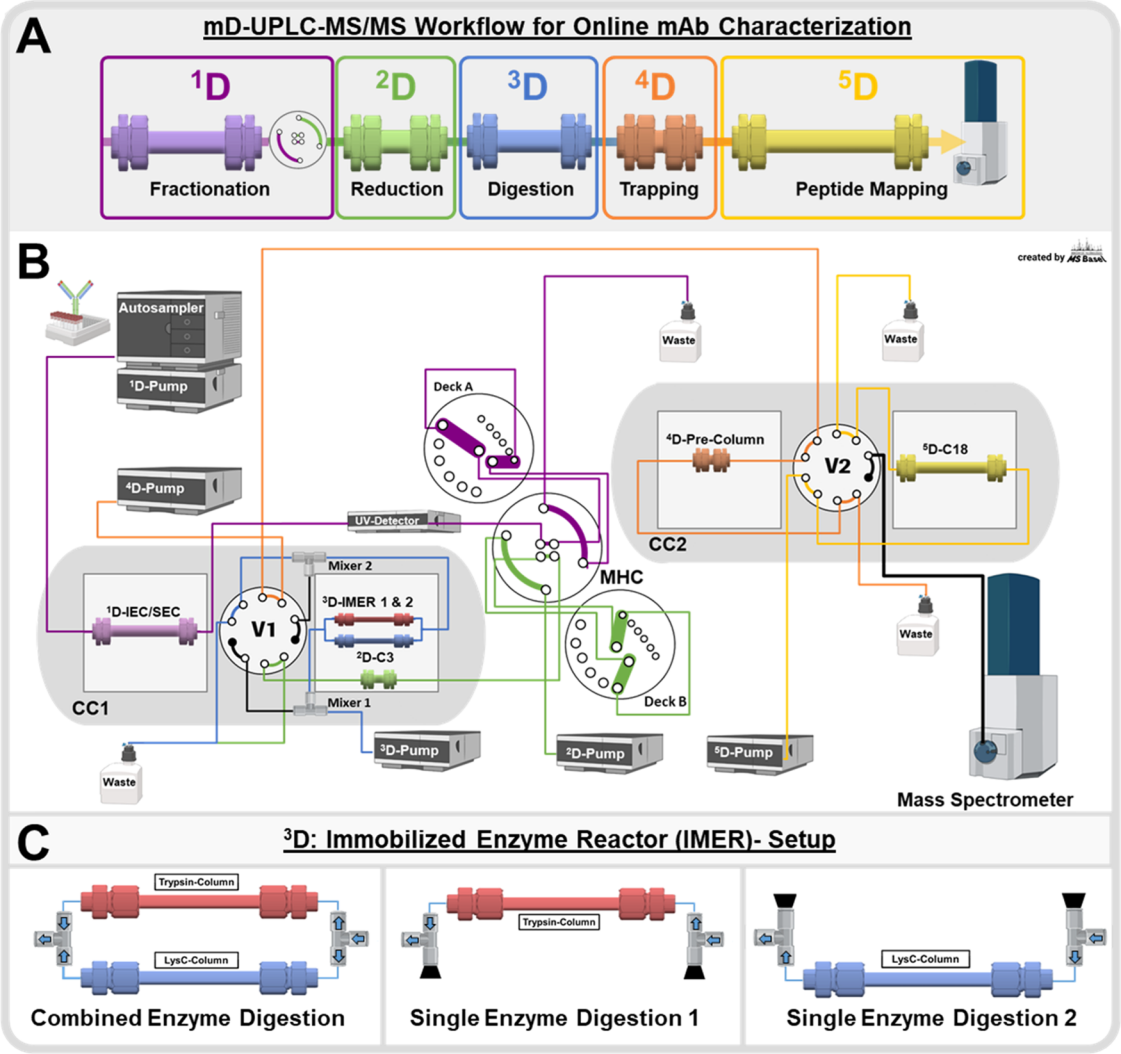
mD-UPLC-MS/MS system setup**:** (A) schematic illustration of the mD-UPLC-MS/MS workflow: first dimension (^1^D): ion-exchange chromatography or size-exclusion chromatography with multiple heart cutting (MHC) online fractionation; second dimension (^2^D): on-column reduction; third dimension (^3^D): on-column digestion by immobilized enzyme reactor 1 and/or 2 (IMER); fourth dimension (^4^D): peptide trapping and desalting; fifth dimension (^5^D): peptide mapping analysis with a high-resolution mass spectrometer (Impact II, Bruker Daltonics). (B) Schematic diagram of the mD-UPLC-MS/MS system with all LC capillaries illustrated as colored lines: MHC = multiple heart cutting valve with loop decks A and B. Each deck incorporates six loops with either 40 or 120 μL volume for online fractionation; CC1/CC2 = column compartment (oven); IMER1 = trypsin-immobilized enzyme reactor; IMER2 = LysC-immobilized enzyme reactor; V1/V2 = 2-position/10-port valve; mixer 1 = bioinert 100 μL static mixer; and mixer 2 = bioinert 150 μL static mixer. (C) Illustration of the three different digestion configurations of the mD-UPLC-MS/MS instrument. The figure was created with BioRender.com.

The schematic setup of our developed mD-UPLC-MS/MS system is shown in Figure 1 and is originally based on the 4D-HPLC/MS system of Gstöttner et al. (2018) but with major improvements. The primary difference between the two instruments includes the implementation of another dimension (mD-UPLC-MS/MS → ^4^D peptide dilution/trapping and ^5^D peptide mapping) that leads to multiple advantages, which are assessed in the following chapters. The main advantages include the usage of long UPLC columns for peptide mapping analysis (≤1300 bar) and a low acetonitrile concentration while peptide trapping, for increased sequence coverage. Despite the implementation of another dimension, we realized to simplify the setup to provide more user friendliness and a reliable system with fewer vulnerabilities for leakage. We achieved this by reducing the number of valves from 3 (4D-HPLC/MS) to 2 (mD-UPLC-MS/MS) by incorporating 2-position/10-port valves instead of 2-position/6-port valves. Additionally, we have replaced the T-pieces of the 4D-HPLC/MS system with biocompatible static mixers for a homogeneous merging of the flows. Furthermore, the mD-UPLC-MS/MS system incorporates a ^2^D binary pump instead of a ^2^D quaternary pump (4D-HPLC/MS). This replacement minimizes the delay volume of the ^2^D-pump and allows us to improve the mD-UPLC performance by reducing the online sample preparation time. Another new feature of the mD-UPLC system includes a versatile LysC and trypsin IMER-setup, thus allowing in addition to the single enzymatic digestion, an in-parallel digestion. In contrast, the 4D-HPLC/MS system uses a single trypsin IMER.

### Comparison of mD-UPLC-MS/MS vs 4D-HPLC/MS

Previous studies with multidimensional LC-MS instruments have shown that there are challenges, such as reduced sequence coverage compared to offline analysis due to incomplete retention of small polar peptides.

**Figure 2:**
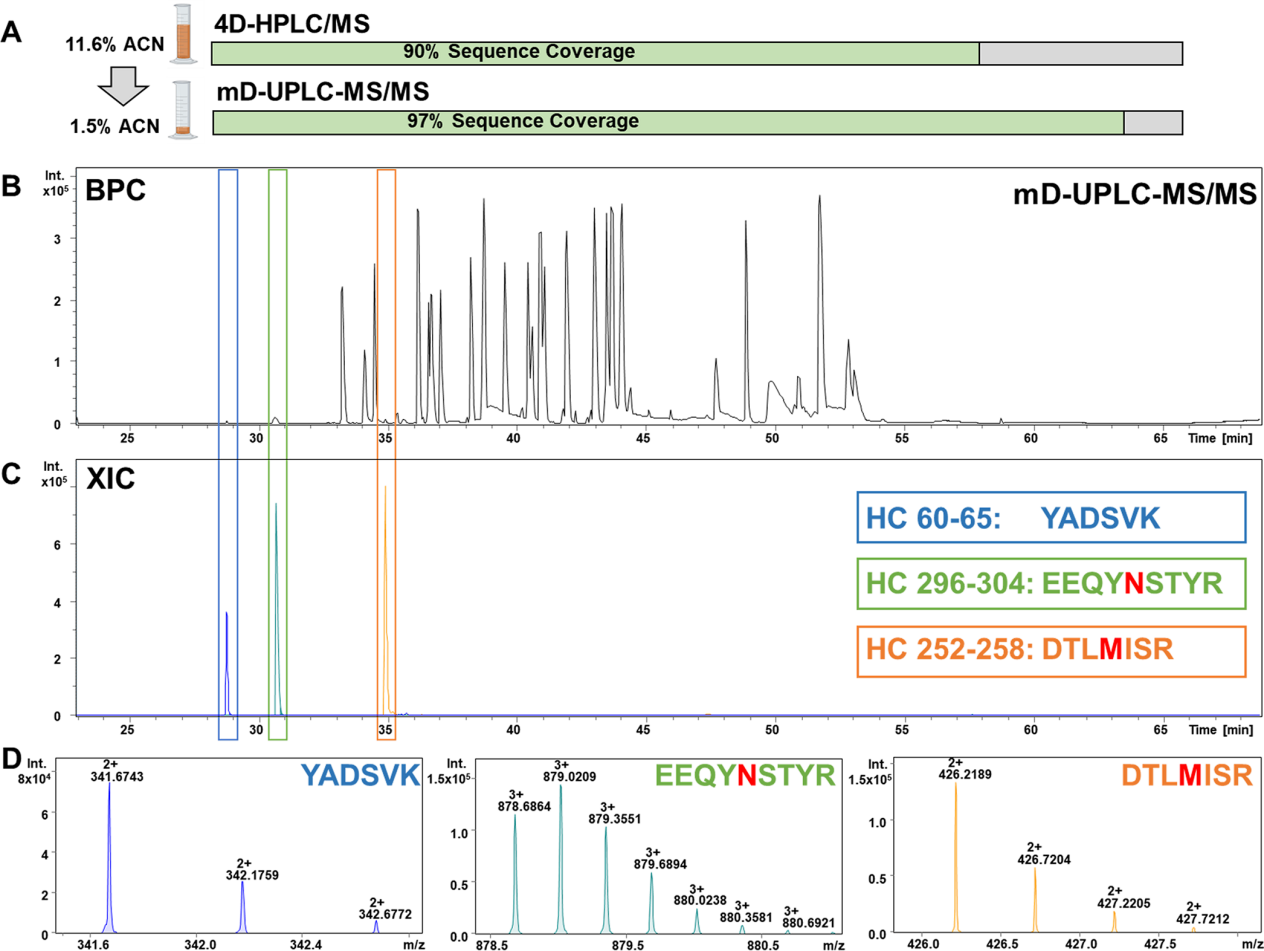
System comparison of mD-UPLC-MS/MS vs 4D-HPLC/MS: (A) sequence coverage comparison of the main peak fraction of Herceptin (trastuzumab) obtained with the 4D-HPLC-MS system published by Gstöttner et al. (2018) and the mD-UPLC-MS/MS system. In addition, the acetonitrile concentration during the peptide trapping step is illustrated for the two systems. (B) Base peak chromatogram (BPC) of the main peak cation-exchange chromatography (CEX) fraction of trastuzumab analyzed with the mD-UPLC-MS/MS instrument. For the analysis, 50 μg was injected into the system and the Agilent InfinityLab Poroshell 120 SB-C18, 3.0 × 5 mm, 1.9 μm precolumn was used in combination with the Agilent InfinityLab Poroshell 120 SB-C18 2.1 × 150 mm, 1.9 μm analytical UPLC column. (C) Extracted ion chromatograms of three small peptides, which are not detected with the 4D-HPLC-MS system by Gstöttner et al. (2018): blue = CDR-H2 peptide YADSVK, green = glycopeptide EEQYNSTYR, and orange = oxidized peptide = DTLMISR. Post-translational modified peptides are highlighted in red. (D) Corresponding MS spectra for the three small peptides: blue: theoretical mass = 681.3334 Da, mass error = 1.03 ppm; green: theoretical mass = 2633.0386 Da, mass error = 0.46 ppm; and orange: theoretical mass = 850.4219 Da, mass error = 1.71 ppm. The figure was in part created with BioRender.com.

To demonstrate the improved performance of our developed mD-UPLC-MS/MS instrument, a comparison with the 4D-HPLC/MS system by Gstöttner et al. (2018) was done, where we prove that we are capable of analyzing the previously not retained peptides. The comparison in Figure 2 shows the base peak chromatogram (BPC) of the mD-UPLC-MS/MS instrument analyzing the main peak fraction (^1^D cation-exchange chromatography (CEX)) of Herceptin (trastuzumab). The results exhibit that the mD-UPLC setup provides a higher sequence coverage (97%) compared to the 4D-HPLC/MS system (90%^14^). In addition, the improved setup enables the identification of the oxidized Met255 (HC, T21: DTLMISR), the CDR-H2 region (HC, T7: YADSVK), the *N*-glycosylated peptide (HC, T23: EEQYNSTYR), and other small peptides. The detection of small peptides and the increased sequence coverage with the mD-UPLC-MS/MS system can be attributed to the low acetonitrile concentration of 1.5% during peptide trapping. In comparison, the 4D-LC-MS system by Gstöttner et al. (2018) provides a high acetonitrile concentration of approximately 11.6%, which results in unretained peptides during the trapping step. The 10-fold increased acetonitrile concentration of the 4D-HPLC/MS system is a result of the in-line connection during online sample preparation of the reducing cartridge (^2^D), the immobilized trypsin column (^3^D), and the peptide mapping column (^4^D). Both the ^2^D and the ^4^D are reversed-phase columns, and a high acetonitrile concentration of 60% is necessary to elute the reduced mAb chains from the ^2^D column. After reduction, the acetonitrile concentration is diluted to 11.6% prior to the immobilized trypsin column. With our new setup, we were able to achieve the low acetonitrile concentration by the ^4^D-pump, which enables an additional dilution step after the trypsin column (^3^D). To ensure high flow rates (^4^D-pump) for effective dilution, the implementation of a low backpressure trapping column, in addition to the analytical column, is necessary. This can be attributed to the system pressure that has to be maintained below the limit of the sensitive trypsin column (^3^D ≤ 170 bar) during digestion. Long C18 columns (50–100 mm, Table 1: pC6, pC7), which are used in recent mD-LC-MS instruments, only enable no or a low dilution rate to ensure moderate ^3^D pressure levels. This results in increased acetonitrile concentration of 6.1–11.6% while peptide trapping. Thus, with recent mD-LC-MS setups, the additional ^4^D dilution step to archive acetonitrile concentrations below 5% is not favorable. In contrast, our setup with small trapping columns (5–30 mm) allows adjusting the acetonitrile concentration to a minimum of 1% by modifying the ^4^D-pump flow rate with the appropriate column (Table 1). The results in Table 1 show that small trapping columns (5 mm, Table 1: pC1–pC3) provide the lowest acetonitrile concentration of 1–1.5% during peptide trapping. With medium trapping columns (30 mm, Table 1: pC4–pC5), acetonitrile concentrations of 2.3–4.5% are obtained. In addition, the capability of adjusting the acetonitrile concentration opens up new opportunities for chromatographic methods, such as hydrophilic interaction chromatography (HILIC), as an increase up to 99% (ACN) is also possible. This potential of the mD-UPLC-MS/MS system will be addressed in another publication.

**Table 1:** Precolumn Pressure and Dilution Comparison^a^

precolumn	ACN [%]	flow. [mL/min]	temp. [°C]	ID [mm]	length [mm]	p. size [μm]
bpC1	1.0	2.20	30	4.6	5	2.7
cpC2	1.2	1.70	30	3.0	5	1.9
dpC3	1.5	1.35	30	2.1	5	1.7
epC4	2.3	0.80	60	4.6	30	2.7
fpC5	4.5	0.25	60	3.0	30	1.7
gpC6	6.1	0.11	60	2.1	100	3.5
hpC7	8.3	0.00	60	2.1	50	1.7

a Comparison of different precolumns used for peptide trapping with the mD-UPLC-MS/MS system. The pressure was measured by the ^3^D-pump during the analysis of the main peak fraction of Herceptin (trastuzumab, 50 μg injection). The mD-UPLC-MS/MS system was operating in the single-enzyme digestion mode with a trypsin IMER installed. The listed flow rates (Flow.) represent the ^4^D-pump flow rate for dilution excluding the ^2^D-pump 0.05 mL/min (50% ACN) and ^3^D-pump 0.25 mL/min (digestion buffer) flow rates. For each column, the highest ^4^D-pump flow rate is listed before the ^3^D-pump exceeds the pressure limit of the ^3^D-trypsin column (<170 bar). In addition, the calculated acetonitrile concentration while peptide trapping on the ^4^D precolumn is listed (ACN). For the small trapping columns with a length of 5 mm, the temperature was set to 30 °C for optimal trapping performance. For longer columns, the temperature (temp.) was set to 60 °C because of the high backpressure at low temperatures. Additionally, the inner diameter (ID), length, and particle size (p. size) are listed for each column.

b pC1: Agilent InfinityLab Poroshell 120 SB-C18 Fast Guards.

c pC2: Agilent InfinityLab Poroshell 120 SB-C18 Fast Guards.

d pC3: Waters ACQUITY UPLC BEH C18 Precolumn.

e pC4: Agilent InfinityLab Poroshell 120 SB-C18 Column.

f pC5: Waters ACQUITY UPLC Peptide BEH C18 Column.

g pC6: Waters XSelect Peptide CSH C18 Column.

h pC7: Waters ACQUITY UPLC Peptide BEH C18 Column.

Goyon and co-workers (2020) took a different approach with their 4D-LC system to reduce the acetonitrile concentration. Instead of an additional dilution, they decreased the ^2^D-pump flow rate to 0.025 mL/min (4D-LC-MS by Gstöttner et al. (2018) = 0.06 mL/min^14^) and used a gradient to elute the reduced mAb chains. This resulted in a final acetonitrile concentration of <6.5% while peptide trapping, which increased the obtained sequence coverage.^16^ With this setup, Goyon et al. (2020) were able to generate reproducible results and detect small polar peptides. Nevertheless, it has to be considered that compared to the mD-UPLC-MS/MS system their method is limited to an acetonitrile concentration of around 6.5%, which could already lead to a loss of hydrophilic peptides. A lower acetonitrile concentration can be beneficial for the characterization of those peptides, for example, if methionine or tryptophan residues are oxidized and the trapping performance is reduced. For this issue, the flexibility of the mD-UPLC-MS/MS system and the possibility of adjusting the acetonitrile concentration down to 1% can be an advantage. Additionally, Goyon et al. (2020) showed that with an increased particle size, C18 columns with 100 mm length can be used for peptide trapping and mapping without damaging the pressure-sensitive trypsin IMER.^16^ Recent publications adopted this approach and demonstrated that the backpressure of these columns is low enough to archive acetonitrile concentrations of approximately 5.5% through dilution.^5,17^ However, with increased particle size, a lower chromatographic resolution and peak capacity is obtained compared to sub 2 μm UPLC columns, which can be used with the mD-UPLC-MS/MS system. In the referred publication, Pot et al. (2021) increased the digestion buffer flow rate to dilute the acetonitrile concentration and was able to characterize small polar peptides.^17^ Compared to our new setup with an additional ^4^D-pump for dilution, this approach has some disadvantages. The higher digestion buffer flow rate results in a shorter digestion time on the immobilized trypsin column, which could lead to less efficient digestion and increased miss cleavage rate.

Besides that, more salt-containing digestion buffer is pumped over the analytical C18 column, which could lead to a more contaminated mass spectrometer. In contrast to recent mD-LC-MS systems, our setup leads to the complete uncoupling of the pressure-sensitive IMER from the full-length analytical peptide mapping column, which offers multiple advantages. Due to the independence from ^3^D pressure limits, this setup allows, to our knowledge, for the first time, a completely free column choice for the peptide mapping analysis by mD-LC-MS instruments. This includes the cutting-edge technology of sub 2 μm UPLC columns with a pressure rating up to 1300 bar for an optimal peptide separation. Thus, with the mD-UPLC-MS/MS system, the established, routine UPLC-MS peptide mapping methods and respective UPLC columns can be selected for online analysis, without compromising inner diameter, length, or particle size. Compared to HPLC columns, the use of UPLC columns can improve both intensity and chromatographic separation, which is a major advantage in PTM characterization.^18−20^ Furthermore, the additional trapping column avoids the high pH and salt-containing digestion buffer by entering the analytical full-length column, which leads to a less contaminated mass spectrometer and improved column lifetime. The more inexpensive guard column also traps undigested protein and impurities, thereby protects the analytical column.

For optimal retention of small polar peptides, we tested different pre-main-column combinations and evaluated them according to the obtained sequence coverage of Herceptin (trastuzumab) (Table 2). The comparison indicates that various C18 stationary phases have different retention capabilities of small peptides and a direct impact on the achieved sequence coverage. In addition, we tested columns with identical stationary phases but with different lengths and inner diameters (IDs). Our results in Table 2 show that the column length and ID have a less significant impact on sequence coverage; thus, small trapping cartridges provide sufficient peptide retention. In contrast, ID, particle size, and length have a huge effect on the column backpressure. A reduction of the ID and particle size causes an increased pressure, while a shorter column results in decreased pressure. For this reason, we recommend small guard cartridges (5–10 mm) with increased ID (3.0–4.6 mm) and low backpressure for optimal peptide trapping. Additional trapping performance of small peptides can be achieved using 30 mm precolumns. Moreover, if a high sequence coverage is required, a low precolumn temperature (30 °C) should be set to increase the trapping performance of less retentive peptides. However, it should be ensured that the temperature of the precolumn is higher than that of the main column during peptide mapping analysis; otherwise, the peptides cannot be refocused on the main column. This is an important factor that should be considered, to use the entire performance of the full-length analytical column to archive an optimal peptide separation. For this reason, we increase the precolumn temperature prior to peptide mapping analysis from 30 to 45 °C to match the main column and improve the chromatography.

**Table 2:** Column Combination Recommendation^a^

precolumn	main column	seq. cov. (%)
Agilent InfinityLab Poroshell 120 SB-C18 2.1 × 5 mm, 1.9 μm	bC1	96
Agilent InfinityLab Poroshell 120 SB-C18 3.0 × 5 mm, 1.9 μm	bC1	97
Agilent InfinityLab Poroshell 120 SB-C18 4.6 × 5 mm, 2.7 μm	bC1	97
Agilent InfinityLab Poroshell 120 SB-C18 4.6 × 30 mm, 2.7 μm	bC1	97
Agilent InfinityLab Poroshell 120 EC-C18 3.0 × 5 mm, 1.9 μm	cC2	93
Agilent AdvanceBio Peptide Mapping 2.1 × 5 mm, 2.7 μm	dC3	96
Agilent AdvanceBio Peptide Mapping 3.0 × 5 mm, 2.7 μm	dC3	96
Agilent AdvanceBio Peptide Mapping 4.6 × 5 mm, 2.7 μm	dC3	96
Waters Atlantis dC18 Column 3.0 μm, 2.1 × 30 mm	eC4	96
Waters ACQUITY UPLC Peptide BEH C18 1.7 μm, 2.1 × 5 mm	eC4	96
Waters ACQUITY UPLC Peptide BEH C18 1.7 μm, 3.0 × 30 mm	eC4	97
Waters ACQUITY UPLC Peptide CSH C18 1.7 μm, 3.0 × 30 mm	fC5	92

a Sequence coverage (seq. cov.) comparison of the main peak CEX fraction of Herceptin (trastuzumab) obtained with the mD-UPLC-MS/MS system with different pre- and main-column combinations. For the analysis, 50 μg of Herceptin (trastuzumab) was injected into the system and the single digestion setup with a trypsin column was used. The data analysis and sequence coverage calculation was accomplished with the PMI-Byos (Byonic) software version 4.0–53 (Protein Metrics Inc.). For the peptide identification, MS/MS spectra were used. The precursor mass tolerance was set to 10 ppm, and a miss cleavage rate of one was permitted.

b C1: Agilent InfinityLab Poroshell 120 SB-C18 2.1 × 150 mm, 1.9 μm.

c C2: Agilent InfinityLab Poroshell 120 EC-C18 2.1 × 150 mm, 1.9 μm.

d C3: Agilent AdvanceBio Peptide Mapping, 2.1 × 150 mm, 2.7 μm.

e C4: Waters ACQUITY UPLC Peptide BEH C18, 1.7 μm, 2.1 × 150 mm.

f C5: Waters ACQUITY UPLC Premier Peptide CSH C18, 1.7 μm, 2.1 × 150 mm.

**Figure 3:**
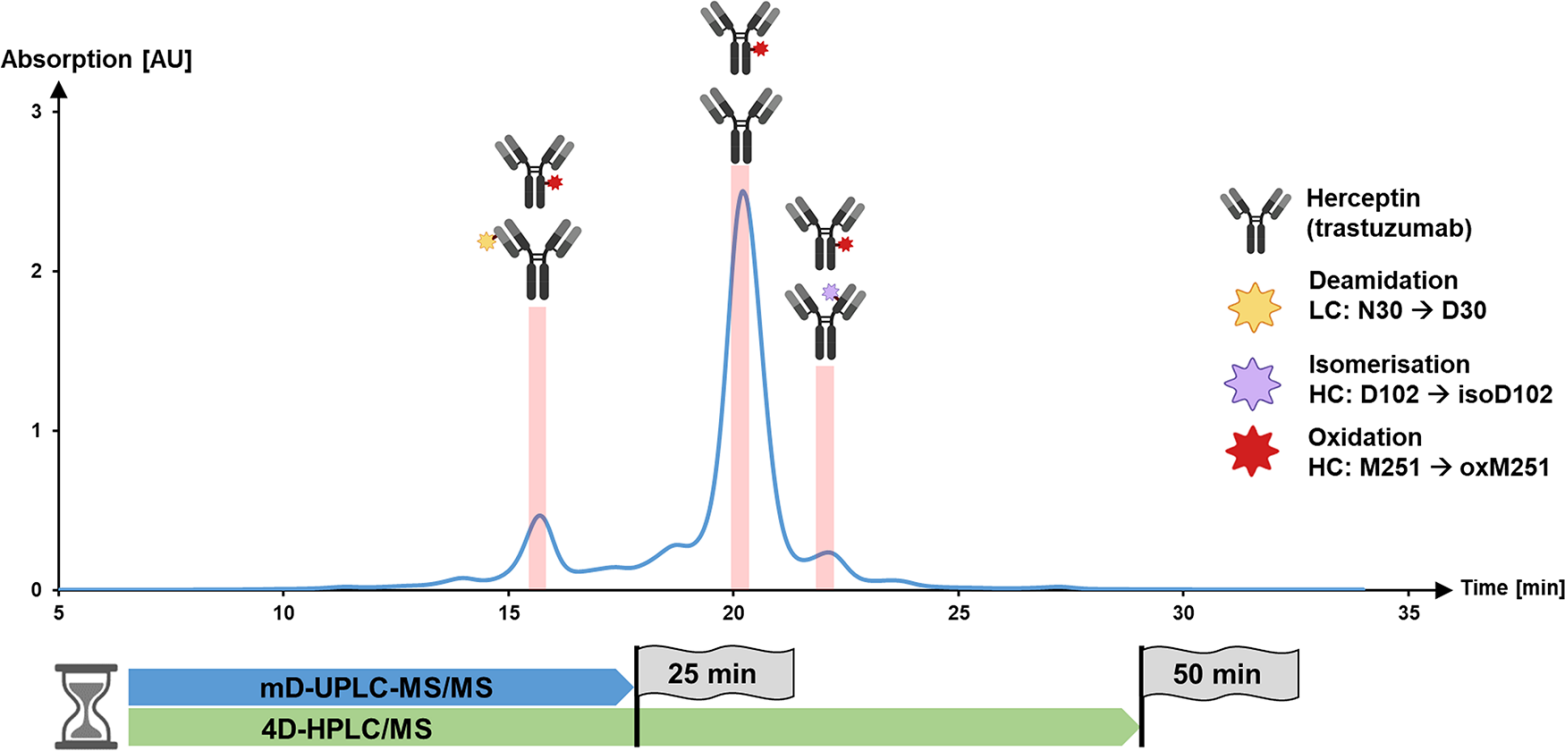
Comparison of the mD-UPLC-MS/MS system with recent mD-LC-MS instruments: illustrated is the CEX chromatogram of Herceptin (trastuzumab) with the mD-UPLC-MS/MS system. The absorbance was measured at 214 nm, and for the analysis, 150 μg of Herceptin (trastuzumab) was injected. The analyzed fractions are indicated as red bars with the identified Herceptin (trastuzumab) species displayed on top. For the indicated Herceptin (trastuzumab) species, the mean of the relative quantification is shown in percent. In addition, the online sample preparation time for each fraction of the mD-UPLC-MS/MS system and the 4D-HPLC/MS instrument is displayed below. The figure was in part created with BioRender.com.

In 2021, Camperi and co-workers evaluated an mD-LC-MS workflow for the extended characterization of mAb charge variants. For further comparison, we characterized the CEX profile of Herceptin (trastuzumab) according to this recent publication.^15^ Therefore, we fractionated, in addition to the main peak, the acidic and basic peak of Herceptin (trastuzumab) (Figure 3). The results in Table 3 indicate that the incorporation of a precolumn does not adversely affect PTM characterization with the mD-UPLC-MS/MS instrument, as all modifications can be characterized with the same accuracy and intensity like recent mD-LC-MS systems (Supporting Table S9). For the acidic peak, we calculated 43.3 ± 0.3% for the deamidation of the light chain asparagine 30 (Table 3, yellow). With recent mD-LC-MS instruments, this modification was determined between 40.4 ± 2.0% and 44.6 ± 0.7%, which is in a comparable range. In addition, the results of the low-abundant modifications (Table 3, black) are also in good agreement with the mD-LC-MS results from Camperi et al. (2021) (Supporting Table S9). However, for the isomerization of the heavy chain (HC) aspartic acid 102, we obtained lower levels compared to the offline characterization and recent mD-LC-MS instruments mentioned by Camperi et al. (2021). With the mD-UPLC-MS/MS instrument, we calculated 35.4 ± 0.1% for the HC-Asp102 isomerization (Table 3, purple), while offline characterization shows higher levels of about 45.3% (Supporting Table S9).^20^ This difference could be explained by the short sample preparation time of only 25 min with the mD-UPLC-MS/MS instrument. In contrast, the offline approach requires long-sample handling (∼24 h), which could induce method-related artifacts, therefore increasing the PTM levels. Furthermore, through the increased retention of small polar peptides with mD-UPLC-MS/MS instrument, we could detect the oxidized methionine 255 of the heavy chain. Our results show that the oxidized HC-Met255 is present in all three fractions (acidic, main, basic) of the CEX with comparable levels between 2.3 ± 0.0% and 3.5 ± 0.0% (Table 3, red). This indicates that the CEX is not suitable to separate the oxidized and the corresponding unmodified Met255 species of Herceptin (trastuzumab). Furthermore, the results in Table 3 show that for the PTM quantification standard deviation values (SD) between 0.0% and 0.4% were obtained. This results in relative standard deviations (RSDs) of under 1% for the primary modifications in the acidic (Deam/Asn30; RSD = 0.7%) and basic peaks (Iso/Asp102; RSD = 0.3%). For the low-abundant modifications (PTM < 4%), low SD values in the range of 0.0–0.4% were also calculated. Due to the low level of these modifications, the calculated RSD values are higher and vary between 0.0% and 12.5%.

**Table 3:**
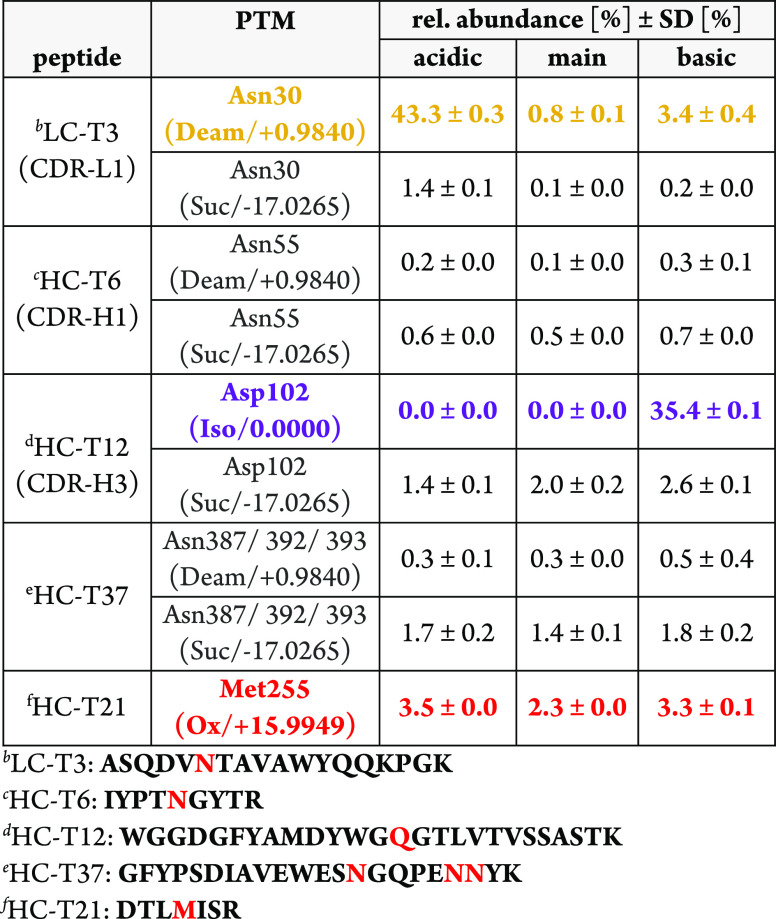
PTM Characterization of Herceptin (trastuzumab) Charge Variants by CEX mD-UPLC-MS/MS^a^

a Relative quantification of the PTM Level from the Herceptin (trastuzumab) CEX fractions acidic, main, and basic obtained by MS/MS peptide mapping analysis with the mD-UPLC-MS/MS instrument. The calculation was performed using PMI-Byos (Byonic) software version 4.0-53 (Protein Metrics Inc.). For the calculation, the area under the curve (AUC) values of the extracted ion chromatograms (XIC) were used. The relative abundance of the modified peptide was calculated based on the XIC-AUC of the modified species divided by the total peak area of the unmodified and modified peptide. Afterward, the arithmetic mean (*n* = 2) and the standard deviation (SD) were calculated for the duplicates.

For the PTMs with very low abundance of ≤0.5%, higher RSD values are observed. The evaluation of these very low-abundant PTMs is more error prone, due to difficulties in setting the integration limits for less intense extracted ion chromatograms (XICs). Nevertheless, the SD and RSD values are in good agreement compared to recent mD-LC-MS systems (Supporting Table S9). Especially, for PTMs > 0.5%, the small RSD values emphasize a good precision and reproducibility for this online approach.

In comparison to recent mD-LC-MS systems, we could also increase the performance and the sample throughput of our instrument by reducing the online sample preparation time by 50% from 50 to 25 min (Figure 3). We realized the significantly shorter time of our developed mD-UPLC-MS/MS system, by replacing the ^2^D quaternary pump with a modified binary pump for on-column reduction. This improvement was achieved, by the potential of our zero-delay volume binary pump with a customized four-channel solvent selection valve. This configuration allows much faster gradients at very low flow rates (50 μL/min) compared to the previously used quaternary pump with a large delay volume of approximately 1 mL.

### Case Study: Characterization of a Bispecific Monoclonal Antibody by mD-UPLC-MS/MS

**Figure 4:**
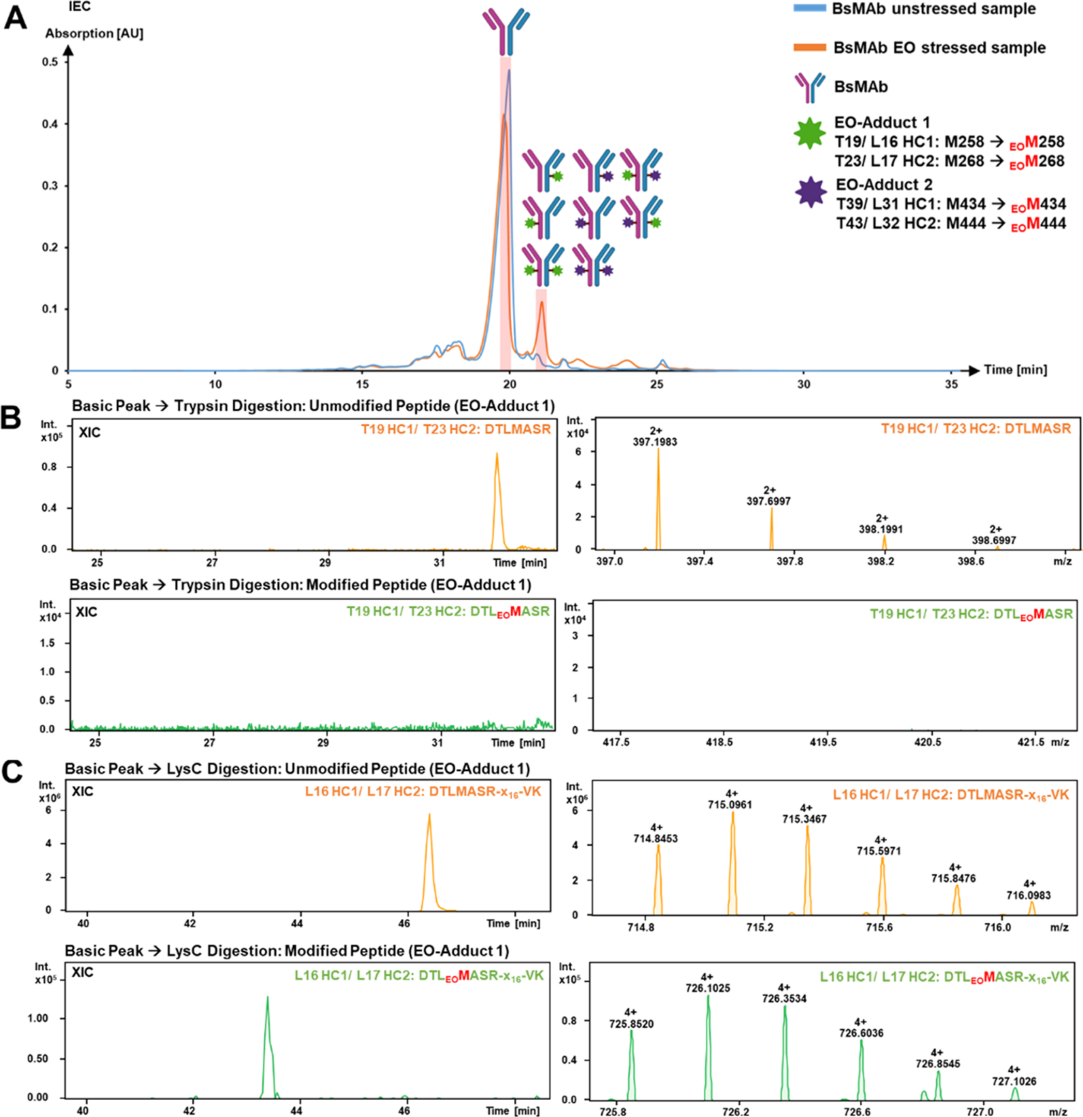
Analysis of ethylene oxide-stressed bispecific antibody with the mD-UPLC-MS/MS system: (A) chromatograms of the ^1^D CEX analyzing a bispecific antibody (provided by F. Hoffmann-la Roche LTD, Basel, Switzerland) with the mD-UPLC-MS/MS system. The absorbance was measured at 280 nm and for the analysis 200 μg of BsMAb was injected. Blue line = BsMAb sample incubated 7 days at 30 °C; orange line = BsMAb sample incubated with 0.01% ethylene oxide for 7 days at 30 °C. The fractionated and analyzed fractions are indicated as red bars with the identified BsMAb species displayed on top. (B) Extracted ion chromatogram and MS spectra of the T19 HC1/T23 HC2 tryptic peptide (orange) and the corresponding ethylene oxide adduct (green). (C) Extracted ion chromatogram and MS spectra of the L16 HC1/L17 HC2 LysC peptide (orange) and the corresponding ethylene oxide adduct (green). The figure was in part created with BioRender.com.

Production is a critical phase in the life cycle of biopharmaceuticals and can lead to unintentional modifications of amino acids. Since undesired PTMs can affect product quality, safety, and efficacy, the production process must be optimized and changes to the molecule need to be monitored. Studies have shown that sterilization of prefilled syringes with ethylene oxide (EO) can lead to methionine, cysteine, and histidine EO adducts.^21,22^ To assess the influence of EO during the filling of a bispecific antibody (BsMAb), forced degradation studies were performed. For the identification of susceptible amino acids, the samples were analyzed by mD-UPLC-MS, and the results were used for further process optimization. Therefore, BsMAb (provided by F. Hoffmann-La Roche LTD, Basel, Switzerland) was incubated for 7 days at 30 °C with 0.01% EO. As a negative control, the unstressed sample was incubated under the same condition without EO. As first dimension of the mD-UPLC-MS/MS system, a CEX was performed incorporating a BioPro IEX-SF, 100 × 4.6 mm, 5 μm column (YMC Europe GmbH). For characterization of unstressed or stressed BsMAb sample, 200 μg was injected and the parameters of Supporting Table S3 were chosen for the ^1^D CEX. The absorbance was detected at 280 nm and the main and basic peaks were fractionated with the MHC valve (Figure 4A). Subsequently, the fractions were processed with the following dimensions and the combined trypsin and LysC digestion setup was used. The ^1^D CEX chromatograms in Figure 4A indicate that the basic peak at approximately 21 min has increased intensity in the EO-stressed sample compared to the unstressed sample. For this reason, we assumed that the EO adducts elute at this time point. To identify the BsMAb adducts, the basic peak, and as a control, the main peak was fractionated and analyzed with the mD-UPLC-MS/MS system. The results in Figure 4B show that only the unmodified tryptic peptide T19/T23 (M258 HC1/M268 HC2) was detected in the basic peak. The EO-adduct (_EO_M258 HC1/_EO_M268 HC2) of the T19/T23 peptide was not detected with the trypsin digestion. Nevertheless, the extracted ion chromatograms (XIC) in Figure 4C show that with the LysC digestion the unmodified peptide and, in addition, the corresponding EO-adduct (L16/L17) were identified. As listed in Table 4, the relative abundance of the EO-adduct 1 was 2.3 ± 0.3% (LysC; RSD = 13.0%) in the basic peak of the EO-stressed sample. The missing identification of the tryptic peptide can be attributed to the sequence of the used BsMAb and the related lack of retention. Compared to Herceptin (trastuzumab), the BsMAb incorporates an alanine instead of an isoleucine (DTLM**I**SR → DTLM**A**SR). This substitution leads to decreased hydrophobicity of the peptide. In addition, the methionine EO-adduct also leads to a more polar peptide species compared to the unmodified peptide, thus less retention on a trapping column. These circumstances further increase the difficulty of characterization with recent multidimensional LC-MS systems and promote the usage of a combined digestion setup. This setup can also be applied to the analysis of methionine oxidation, thus enhancing the characterization by LysC digestion. Due to the additional LysC digestion, longer peptides can be generated compared to trypsin digestion, if they end on an arginine. In general, the enlarged peptide length by LysC digestion increases retention on the trapping column and simplifies characterization. Furthermore, the combined digestion can generate two distinct peptides for the same modified amino acid, therefore supporting the identification of PTMs. The dual identification can be observed for the second EO-adduct that we found for the basic peak fraction of the EO-stressed sample. As listed in Table 4, the EO-adduct 1 was identified as trypsin and LysC peptide, which increases the chance of a true positive identification. In addition, the relative abundance of the tryptic and LysC EO-adduct 2 were comparable with values of 3.3 ± 0.0% (Trypsin; RSD = 0.0%) and 3.7 ± 0.1% (LysC; RSD = 2.7%). The minor difference can be explained by the varying miss cleavage rate of the two IMER columns, which can affect relative quantification. The low SD and RSD values of the triplicates for the PTM quantification show that the mD-UPLC-MS/MS system provides reproducible and reliable results over multiple injections. Moreover, the combined digestion setup increased the obtained sequence coverage of BsMAb from 95 to 98%. Nevertheless, it should be mentioned that the combined digestion setup is only beneficial if the mAb concentration of the ^1^D fraction is high enough. Otherwise, the intensity while peptide mapping can be too low for proper characterization of PTMs. Additionally, we would recommend to use long analytical C18 UPLC columns (length ≤ 150 mm) for the combined digestion setup for an optimal trypsin and LysC peptide separation and less coelution. This should be considered because of the increased amount of unique peptides in a parallel digestion setup compared to the single enzymatic digestion.

**Table 4:**
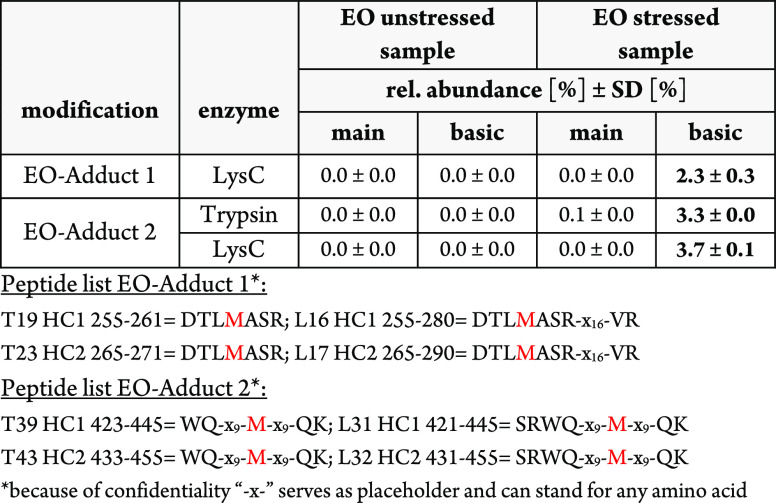
Results of the Unstressed and Ethylene Oxide-Stressed BsMAb Samples with the mD-UPLC-MS/MS System^a^

a For the characterization of susceptible amino acids for the ethylene oxide adduct formation, stressed and unstressed samples were analyzed with the mD-UPLC-MS/MS system incorporating a ^1^D CEX. The stressed sample was incubated for 7 days at 30 °C with 0.01% ethylene oxide, while the unstressed sample was incubated under the same conditions without ethylene oxide. The main and basic peaks of each sample were analyzed in triplicates, and the relative abundance was calculated with the area under the curve (AUC) values of the extracted ion chromatograms (XIC). The relative abundance of the modified peptide was calculated based on the XIC-AUC of the modified peptide divided by the total peak area of the unmodified and modified peptide. The arithmetic mean (*n* = 3) of the relative abundance is listed together with the standard deviation (SD). For the analysis, the combined enzyme digestion setup (trypsin, LysC) of the mD-UPLC-MS/MS system was used. The data analysis was accomplished with the PMI-Byos (Byonic) software version 4.0–53 (Protein Metrics Inc.) and two separate workflows for trypsin and LysC. For the peptide identification, MS/MS spectra were used. The precursor mass tolerance was set to 10 ppm, and a miss cleavage rate of one was permitted.

## Conclusions

Multidimensional LC-MS instruments enable rapid and automated characterization of biopharmaceuticals with the ability to online fractionate from various ^1^D chromatographic methods. Depending on the first dimension, these versatile systems lead to the possibility to support in multiple phases of mAb process, drug, and formulation development. Nevertheless, current mD-LC-MS instruments have limitations; therefore, they are in a continuous state of development. In this study, we addressed the major challenges of recent mD-LC-MS systems and unveiled our latest solutions. We improved the entire system setup and evolved the multidimensional HPLC system to a state-of-the-art UPLC system. This enhanced design allows a free column selection for peptide mapping analysis including UPLC columns (≤1300 bar), which can increase peak performance and improve mAb characterization. Additionally, this allows the transfer of established, routine UPLC-MS peptide mapping methods onto the mD-UPLC-MS/MS system. Therefore, an optimized method can be used and a convenient comparison between the on- and offline data is possible. Furthermore, this setup allows the implementation of a novel in-parallel IMER (trypsin; LysC) digestion setup. This setup was incorporated without compromising analysis time or exceeding the IMER pressure limits (<170 bar). We could demonstrate in our case study that this enables the identification of a very low retentive ethylene oxide-modified peptide, which can occur during mAb production. Another advantage of the parallel digestion setup is that a unique trypsin and a LysC peptide can be received. This increases the likelihood of PTM characterization and the obtained sequence coverage. With the increasing number of more complex bispecific mAbs, in-parallel digestion can be a significant advantage for PTM characterization. To enhance the detection of low retentive peptides and increase the obtained sequence coverage, we incorporated an acetonitrile dilution step prior peptide trapping. Compared to recent mD-LC-MS systems, this approach enables to adjust the acetonitrile concentration to a minimum of 1%, which is equal to the offline method. This study further demonstrates the value of this system for the pharmaceutical industry as all 12 measurements can be accomplished fully automated in under 18 h. Given these improvements, the introduced system provides a fast and reliable methodology and increases the efficiency of routine mAb characterization.

## Supporting Information Available

Additional experimental details, including reagents (Table S1); mD-UPLC-MS/MS instrument modules (Table S2); gradients and parameters of LC-dimensions (Tables S3–S7); mass spectrometer parameters (Table S8); and for comparison, the results of the charge variant analysis from recent mD-LC-MS systems and from an offline approach are listed (Table S9) (PDF)
