# Supplementary material for: mD-UPLC-MS/MS: Next Generation of mAb Characterization by Multidimensional Ultraperformance Liquid Chromatography-Mass Spectrometry and Parallel On-Column LysC and Trypsin Digestion
Source: Anal Chem. 2022 May 12;94(23):8136–45. doi: 10.1021/acs.analchem.1c04450 (PMC9201819; doi:10.1021/acs.analchem.1c04450)
Supplement: Supplementary file 1 — ac1c04450_si_001.pdf [file ac1c04450_si_001.pdf]

Supporting Information for:

mD-UPLC-MS/MS: Next Generation of mAb Characterization by Multidimensional Ultraperformance Liquid Chromatography-Mass Spectrometry and Parallel On-column LysC and Trypsin Digestion

Saban Oezipek†, Sina Hoelterhoff†, Simon Breuer†, Christian Bell†, Anja Bathke†*

†Pharma Technical Development, F. Hoffmann-La Roche, Grenzacherstrasse 124, 4070 Basel, Switzerland

*Corresponding author: Anja Bathke, anja.bathke@roche.com

Table of Contents

Table S1: Reagent List 2

Table S2: Modules of the mD-UPLC-MS/MS System 3

Table S3: ^{1}D Cation-Exchange Chromatography Gradient and Parameters 4

Table S4: ^{2}D Online Reduction Gradient and Parameters 5

Table S5: ^{3}D Online Digestion Gradient and Parameters 6

Table S6: ^{4}D Peptide Trapping Gradient and Parameters 7

Table S7: ^{5}D Peptide Mapping Gradient and Parameters 8

Table S8: Parameters of the mD-UPLC-MS/MS Mass Spectrometer 9

Table S9: Herceptin CEX Characterization by Schmid et al., (2018)[1] and Camperi et al., (2021)[2] 10

Table S1: Reagent List

Reagent	Manufacturer	CAS No.
Sodium dihydrogen phosphate monohydrate	MERCK KGaA, Darmstadt, Germany	10049-21-5
di-Sodium hydrogen phosphate	MERCK KGaA, Darmstadt, Germany	7558-79-4
Calcium chloride anhydrous Powder	MERCK KGaA, Darmstadt, Germany	10043-52-4
Tris(hydroxymethyl) aminomethane (TRIS)	MERCK KGaA, Darmstadt, Germany	77-86-1
Tris(2-carboxyethyl)phosphin (TCEP)	MERCK KGaA, Darmstadt, Germany	51805-45-9
Acetonitrile (ACN, HPLC-grade)	MERCK KGaA, Darmstadt, Germany	75-05-8
Sodium chloride	Fluka Missouri, USA	7647-14-5
Formic acid (FA)	Fluka Missouri, USA	64-18-6
N,N-Bis(2-hydroxyethyl)-2-aminoethanesulfonic acid (BES)	MERCK KGaA, Darmstadt, Germany	10191-18-1
N,N-Bis(2-hydroxyethyl)-2-aminoethanesulfonic acid sodium salt (BES sodium salt)	MERCK KGaA, Darmstadt, Germany	66992-27-6
Purified Water (Milli-Q H_2O)	Merck Millipore (Merck KGaA, Darmstadt, Germany)<br>Milli-Q Advantage A10 system	

Table S2: Modules of the mD-UPLC-MS/MS System

The mD-UPLC-MS/MS system is configured as two instruments within the OpenLab software package (ChemStation). The communication between the two instruments was performed by a custom made macro “valve event plugin” from ANGI (Gesellschaft für angewandte Informatik, Karlsruhe, Germany).

Instrument 1 (^{1}D/^{5}D)		
**Module**	**Product**	**Product No.**
Autosampler	1260 HiP Bio ALS	G5667A
^{1}D-Pump	1290 binary pump	G4220A
^{5}D-Pump	1290 binary pump	G4220A
Multiple Heart Cutting Valve (MHC)	2-position/4-port duo-valve	G4236A (MHC-kit)<br>5067-4214 (valve)
Loop Deck A with 6 sampling loops (120 µL Volume)	6-position/14-port valve	G4242A (MHC upgrade kit)<br>5067-4142 (valve)
Loop Deck B with 6 sampling loops (120 µL Volume)	6-position/14-port valve	G4242A<br>5067-4142 (valve)
UV detector 1	1260 VWD	G1314F
**Instrument 2 (^{2}D/^{3}D/^{4}D)**		
**Module**	**Product**	**Product No.**
^{2}D-Pump	1290 binary pump	G4220A
^{3}D-Pump	1260 quaternary pump	G1311B
^{4}D-Pump	1260 quaternary pump	G1311B
Column compartment 1 (CC1)<br>installed 2-position/10-port valve (800 bar)	1290 TCC	G1316C (CC1)<br>5067-4283 (valve)
Column compartment 2 (CC2)<br>installed 2-position/10-port valve (1300 bar)	1290 MCT	G1314F (CC2)<br>5067-4240 (valve)

Table S3: [1]D Cation-Exchange Chromatography Gradient and Parameters

Product specific [1]D CEX parameters and gradients are listed for Herceptin (trastuzumab) and the bi-specific mAb (BsMAb). For the Herceptin CEX a Thermo Scientific ProPac™ WCX-10 Analytical, 4 x 250mm column was used at 25°C. The absorbance was measured with the VWR detector at 214 nm. The BsMAb CEX was performed with a YMC BioPro IEX-SF, 100 x 4.6 mm, 5 µm column at 40°C and the absorbance was measured at 280 nm.

**[1]D-Pump Cation Exchange Chromatography Herceptin (trastuzumab)**				
**Time [min]**	**Flowrate [mL/min]**	**Eluent A [%] (10 mM sodium phosphate, pH 7.5)**	**Eluent B [%] (10 mM sodium phosphate, 100 mM NaCl pH 7.5)**	**Comment**
0.00	0.8	85	15	CEX and peak fractionation by multiple heart cutting
30.00		45	55	
35.00		45	55	
36.00		0	100	Wash to reduce carryover
44.00		0	100	
45.00		85	15	Column equilibration
55.00		85	15	

**[1]D-Pump Cation Exchange Chromatography BsMAb**				
**Time [min]**	**Flowrate [mL/min]**	**Eluent A [%] (20 mM BES, pH 6.8)**	**Eluent B [%] (20 mM BES, 500 mM NaCl, pH 6.8)**	**Comment**
0.00	0.8	98	2	CEX and peak fractionation by multiple heart cutting
5.00		98	2	
35.00		85	15	
35.10		0	100	Wash to reduce carryover
40.00		0	100	
40.10		98	2	Column equilibration
50.00		98	2	

Table S4: ^{2}D Online Reduction Gradient and Parameters

The reduction was performed on a Poroshell 300SB-C3 2.1 x 12.5 mm, 5.0 µm (Agilent Technologies) cartridge at 40°C.

^{2}D-Pump On-Column Reduction						
**Time [min]**	**Flowrate [mL/min]**	**Eluent A1 [%] (20 mM TCEP, 5% ACN in H_2O)**	**Eluent B1[%] (0.1% FA, 5% ACN in H_2O)**	**Eluent A2 [%] (0.1% FA in H_2O)**	**Eluent B2 [%] (0.1% FA in ACN)**	**Comment**
0.00	0.50	0	100	-		Start instrument 2 (^{2}D/^{3}D/^{4}D)
0.01		100	0	-		^{2}D column trapping and on-column reduction
4.00		100	0	-		
4.01	2.00	-		85	15	Solvent selection valve switch A1/ B1 → A2/ B2 and wash
7.00		-		85	15	
7.01	0.50	-		50	50	Valve 1 switch ^{2}D→^{3}D →^{4}D
7.25		-		50	50	Elution of reduced mAbs
8.00		-		50	50	
8.01		-		100	0	
8.50		-		100	0	
8.51		-		50	50	
11.50		-		50	50	
11.51		-		100	0	
12.00		-		100	0	
12.01		-		50	50	
15.00		-		50	50	
15.01		-		100	0	
15.50		-		100	0	
15.51		-		50	50	
18.50		-		50	50	
18.51		-		100	0	
19.00		-		100	0	
19.01		-		50	50	
22.00		-		50	50	
22.01		-		50	50	Valve 1 switch ^{2}D→ waste
23.00	1.00	-		5	95	Wash to reduce carryover
23.01		-		95	5	
24.00		-		5	95	
24.01		-		95	5	
25.00		-		5	95	
25.01		-		95	5	
26.00		-		5	95	
26.01		-		95	5	
27.00		-		5	95	
27.01		-		95	5	
28.00		-		5	95	
28.01		-		95	5	
29.00		-		5	95	
29.01		-		95	5	
30.00		-		5	95	
35.00		-		5	95	
35.01	0.5	50	50	-		Solvent selection valve switch A2/ B2 → A1/ B1
40.00		50	50	-		
40.01		0	100	-		Column equilibration
60.00		0	100	-		

Table S5: ^{3}D Online Digestion Gradient and Parameters

For the online digestion a custom made LysC (2.1 x 100 mm, Perfinity Biosciences) and/ or a trypsin (2.1 x 100 mm, Perfinity Biosciences) immobilized enzyme reactor (IMER) was used at 40°C.

^{3}D-Pump On-Column Digestion				
**Time [min]**	**Flowrate [mL/min]**	**Eluent A [%] (50 mM TRIS, 10 mM $CaCl_2$, pH 8.5)**	**Eluent B [%] (ACN)**	**Comment**
0.00	0.25	100	0	Start instrument 2 (^{2}D/^{3}D/^{4}D)
0.01	1.50	50	50	Wash to reduce carryover
4.00		50	50	
4.01		100	0	
6.00		100	0	
6.01	0.25	100	0	
7.01		100	0	Valve 1 switch ^{2}D→^{3}D →^{4}D
7.02		100	0	Digestion of reduced mAbs
22.00		100	0	
22.01		100	0	Valve 1 switch ^{2}D→^{3}D →^{4}D
25.00		100	0	Wash to reduce carryover
25.01	1.50	50	50	
30.00		50	50	
30.01		100	0	
31.00		100	0	
31.01		50	50	
32.00		50	50	
32.01		100	0	
33.00		100	0	
33.01		50	50	
34.00		50	50	
34.01		100	0	
35.00	0.25	100	0	Column equilibration

Table S6: ⁴D Peptide Trapping Gradient and Parameters

The peptide trapping was performed on a precolumn, which matches with the main column. For Herceptin an InfinityLab Poroshell 120 SB-C18 3.0x 5 mm, 1.9 μm (Agilent Technologies) precolumn was used at 30°C. For the BsMAb analysis an ACQUITY UPLC BEH C18 2.1x 5mm, 1.7 μm (Waters Corporation) precolumn was used at 30°C and switched to 45°C one minute prior peptide mapping analysis (24 min).

^{4}D-Pump Peptide Trapping						
**Time [min]**	**Flowrate [mL/min]**	**Eluent A [%] (0.1% FA in H_2O)**	**Eluent B [%] (0.1% FA in ACN)**	**Eluent C [%] (H_2O)**	**Eluent D [%] (ACN)**	**Comment**
0.00	0.25	95	5	0	0	Start instrument 2 (^{2}D/^{3}D/^{4}D)
0.01	1.00	95	5	0	0	Wash to reduce carryover
1.00		5	95	0	0	
2.00		5	95	0	0	
2.01		95	5	0	0	
3.00		5	95	0	0	
4.00		5	95	0	0	
4.01		99	1	0	0	
5.00		0	0	100	0	
7.01	1.35 – 2.20 (depending on precolumn)	0	0	100	0	Valve 1 switch ^{2}D→^{3}D →^{4}D
7.02		0	0	100	0	^{4}D column trapping
22.00		0	0	100	0	
22.01		0	0	99	1	Valve 1 switch ^{4}D → waste
22.02		0	0	99	1	Wash to reduce salt concentration
25.00		0	0	99	1	
25.01	0.10	95	5	0	0	Valve 2 switch ^{4}D→ ^{5}D → MS
59.00		95	5	0	0	Valve 2 switch ^{4}D→ waste
59.01	0.25	95	5	0	0	Column equilibration

Table S7: ^{5}D Peptide Mapping Gradient and Parameters

The peptide mapping analysis was performed on an UPLC-column, depending on the analyzed mAb. For Herceptin an InfinityLab Poroshell 120 SB-C18 2.1 x 150 mm, 1.9 µm (Agilent Technologies) UPLC-column was used at 40°C. For the BsMAb analysis an Waters ACQUITY UPLC Peptide BEH C18 Column, 300Å, 1.7 µm, 2.1 mm X 150 mm (Waters Corporation) UPLC-column was used at 40°C. For both columns a flowrate of 0.4 mL/min was used.

**^{5}D-Pump Peptide Mapping Analysis**			
**Time [min]**	**Eluent A [%] (0.1% FA in H_2O)**	**Eluent B [%] (0.1% FA in ACN)**	**Comment**
0.00	95	5	Start instrument 2 (^{2}D/^{3}D/^{4}D)
0.01	95	5	Wash to reduce carryover
2.00	5	95	
3.00	5	95	
3.01	95	5	
5.00	5	95	
6.00	5	95	
6.01	95	5	
8.00	5	95	
9.00	5	95	
9.01	95	5	
11.00	5	95	
12.00	5	95	
15.00	99	1	
25.01	99	1	Valve 2 switch ^{4}D→ ^{5}D → MS
65.00	60	40	Peptide mapping analysis
68.00	40	60	
69.00	5	95	
70.00	5	95	
70.01	95	5	Wash to reduce carryover
71.00	5	95	
72.00	5	95	
72.01	95	5	
73.00	5	95	
74.00	5	95	
74.01	95	5	Valve 2 switch ^{4}D→ waste ^{5}D → waste
75.00	95	5	Column equilibration

Table S8: Parameters of the mD-UPLC-MS/MS Mass Spectrometer

Parameters of the Impact II high resolution mass spectrometer from Bruker Daltonics which is coupled to the mD-UPLC system.

**Mass Spectrometer Parameters**	
Spray voltage	4.5 kV
End plate offset	500 V
Nebulizer	2.0 bar
Dry gas flow	11.0 L/min
Dry temperature	220°C
MS mode	positive
Scan range	150 – 2000 m/z
Spectra rate	2.00 Hz
Fragmentation	Collision-induced dissociation (CID)
MSMS	Auto, cycle time 3.0 sec

Table S9: Herceptin CEX Characterization by Schmid et al., (2018)[1] and Camperi et al., (2021)[2]

Relative quantification of the PTM level from the Herceptin CEX fractions acidic, main and basic obtained by MS/MS peptide mapping analysis. Method Offline: The CEX fractionation and peptide mapping analysis was performed with the offline approach without mD-LC/MS instruments by Schmid et al., (2018)[1]. Method Lab 1-3: The CEX fractionation and peptide mapping analysis was performed with three different mD-LC/MS instruments by Camperi et al., (2021)[2]. The standard deviation (SD) from triplicates is listed for Camperi et al., (2021)[2].

Adapted with permission from Camperi, J.; Grunert, I.; Heinrich, K.; Winter, M.; Oezipek, S.; Hoelterhoff, S.; Weindl, T.; Mayr, K.; Bulau, P.; Meier, M., Inter-laboratory Study to Evaluate the Performance of Automated Online Characterization of Antibody Charge Variants by Multi-Dimensional LC-MS/MS. Talanta 2021, 122628. Copyright 2021 Elsevier

Tryptic Peptide	PTM Position	Method	Relative Quantification of PTMs by MS/MS [%] ± SD [%]		
			Acidic	Main	Basic
LC-T3 (CDR-L1) **ASQDVNTAVAWYQQKPGK**	Asn30 (Deam/+0.9840)	Offline	49.5	5.4	4.4
		Lab1	42.0 (±0.4)	2.5 (±0.2)	2.4 (±0.3)
		Lab2	44.6 (±0.7)	2.9 (±0.2)	2.8 (±0.2)
		Lab3	40.4 (±2.0)	2.5 (±1.1)	2.4 (±0.8)
	Asn30 (Suc/-17.0265)	Offline	0.2	0.2	0.2
		Lab1	0.6 (±0.2)	0.5 (±0.1)	0.45 (±0.1)
		Lab2	0.7 (±0.1)	0.9 (±0.4)	0.9 (±0.1)
		Lab3	1.0 (±0.7)	1.3 (±0.4)	1.0 (±0.2)
HC-T6 (CDR-H1) **IYPTNGYTR**	Asn55 (Deam/+0.9840)	Offline	1.4	1.0	1.1
		Lab1	0.5 (±0.1)	0.3 (±0.1)	0.1 (±0.1)
		Lab2	0.8 (±0.1)	0.7 (±0.1)	0.7 (±0.1)
		Lab3	0.8 (±0.1)	0.7 (±0.1)	0.8 (±0.2)
	Asn55 (Suc/-17.0265)	Offline	3.9	4.0	3.9
		Lab1	1.3 (±0.3)	1.4 (±0.3)	1.4 (±0.4)
		Lab2	1.8 (±0.1)	1.7 (±0.1)	1.6 (±0.3)
		Lab3	1.3 (±0.1)	1.4 (±0.1)	1.4 (±0.3)
HC-T12 (CDR-H3) **WGGDGFYAMDYWGQGTLVTVSSASTK**	Asp102 (Iso/0.0000)	Offline	4.6	6.0	45.3
		Lab1	0.0	0.0	59.8 (±1.2)
		Lab2	0.0	0.0	64.7 (±1.9)
		Lab3	0.0	0.0	65.2 (±1.2)
	Asp102 (Suc/-17.0265)	Offline	2.7	2.7	2.7
		Lab1	1.8 (±0.1)	2.0 (±0.1)	1.2 (±0.2)
		Lab2	1.7 (±0.2)	1.5 (±0.2)	1.7 (±0.2)
		Lab3	2.0 (±0.1)	2.3 (±0.1)	1.6 (±0.3)
HC-T37 **GFYPSDIAVEWESNGQPENNYK**	Asn387/ 392/ 393 (Deam/+0.9840)	Offline	1.6	1.6	0.9
		Lab1	0.5 (±0.1)	0.5 (±0.2)	0.4 (±0.1)
		Lab2	0.9 (±0.1)	0.8 (±0.1)	0.8 (±0.1)
		Lab3	0.6 (±0.1)	0.6 (±0.1)	0.7 (±0.1)
	Asn387/ 392/ 393 (Suc/-17.0265)	Offline	1.5	1.6	1.5
		Lab1	1.8 (±0.2)	1.8 (±0.2)	1.5 (±0.1)
		Lab2	1.8 (±0.4)	2.4 (±0.5)	1.6 (±0.3)
		Lab3	1.9 (±0.1)	2.2 (±0.4)	2.0 (±0.3)

[1] Schmid I, Bonnington L, Gerl M, Bomans K, Thaller AL, Wagner K, Schlothauer T, Falkenstein R, Zimmermann B, Kopitz J, Hasmann M, Bauss F, Haberger M, Reusch D, Bulau P. Assessment of susceptible chemical modification sites of trastuzumab and endogenous human immunoglobulins at physiological conditions. Commun Biol. 2018;1:28

[2] Camperi, J.; Grunert, I.; Heinrich, K.; Winter, M.; Oezipek, S.; Hoelterhoff, S.; Weindl, T.; Mayr, K.; Bulau, P.; Meier, M., Inter-laboratory Study to Evaluate the Performance of Automated Online Characterization of Antibody Charge Variants by Multi-Dimensional LC-MS/MS. Talanta 2021, 122628.
